# Acceptability and perceived impact of a mental health and disability programme in Ghana

**DOI:** 10.4102/ajod.v14i0.1659

**Published:** 2025-08-22

**Authors:** Lionel Sakyi, Lyla Adwan-Kamara, Kenneth A. Ae-Ngibise, Crick Lund

**Affiliations:** 1 Ghana Somubi Dwumadie (Ghana Participation Programme), Accra, Ghana; 2 Research and Development Division, Ghana Health Service, Kintampo Health Research Centre, Bono East Region, Ghana; 3 Centre for Global Mental Health, Health Service and Population Research Department, Institute of Psychiatry, Psychology and Neuroscience, King’s College London, London, United Kingdom; 4 Department of Psychiatry and Mental Health, Alan J Flisher Centre for Public Mental Health, University of Cape Town, Cape Town, South Africa

**Keywords:** disability inclusion, mental health, process evaluation, Ghana, user-led, Theory of Change

## Abstract

**Background:**

Ghana Somubi Dwumadie aimed to improve wellbeing of people with disabilities, including people with mental health conditions, through four pillars: strengthening policies and systems; scaling up integrated, accessible mental health services; reducing stigma and discrimination; and generating evidence to inform policy and practice. Despite these efforts, its implementation has not been comprehensively evaluated.

**Objectives:**

To assess the programme’s acceptability and perceived impact from the perspective of key stakeholders, including government, civil society organisations, and grantees.

**Methods:**

A process evaluation, guided by the programme’s Theory of Change, involved in-depth interviews with 32 stakeholders from programme partners, civil society and government. Document reviews supplemented data collection, and thematic analysis identified key insights.

**Results:**

Stakeholders highlighted the programme’s technical assistance as crucial for strengthening advocacy and policy leadership in mental health. The integration of mental health services into primary care and the involvement of traditional leaders to reduce stigma were noted successes. Challenges included funding instability, gaps between advocacy and service delivery and limited control over implementing key policy reforms.

**Conclusion:**

Ghana Somubi Dwumadie made significant strides in disability inclusion and mental health care through its multi-sectoral, user-led approaches. Addressing challenges like sustainable funding and service delivery will be critical for ensuring lasting impact and scalability.

**Contribution:**

This study underscores the impact of user-led, multi-sectoral approaches in reducing stigma, scaling services, and empowering people with disabilities in resource-limited settings, while addressing challenges and strategies for disability-inclusive programming.

## Introduction

It is estimated that 16% of the world’s population experience significant disability (WHO 2022). The number of people with disabilities continues to increase, particularly in low- and middle-income countries (LMICs), where it is estimated to be between 10% and 15% of the total population with almost 80% living in sub-Saharan Africa (Mitra & Yap 2021). In Ghana, people with disabilities constitute 8% of the country’s population (Ghana Statistical Service 2024). People with disabilities face unique daily challenges that impact several aspects of their lives. Challenges such as physical impairments, social prejudices and societal limitations prevent people with disabilities from accessing opportunities and resources equally (WHO 2020).

Despite Ghana’s ratification of the United Nation Convention on the Rights of Persons with Disabilities (CRPD), its participation in the African Decade of Persons with Disabilities and the enactment of the *Persons with Disability Act 2006 (Act 715)*, individuals with disabilities in Ghana continue to face significant barriers to equal societal participation (CRPD 2024). Addressing disability, including mental health-related disability, is crucial in achieving sustainable development as it ensures that no one, particularly more disadvantaged people in society, are left behind. The Sustainable Development Goals (SDGs) and its agenda of ‘leave no one behind (LNOB)’ emphasise the importance of including people with disabilities in mainstream development (Jolley et al. 2018). Despite the efforts of the SDGs, real impact and progress for people with disabilities has been minimal (Opoku et al. 2019). In response to these systemic challenges, the Ghana Somubi Dwumadie programme was launched as an initiative aimed at improving the well-being of people with disabilities, including those with mental health conditions.

With funding from the UK’s Foreign, Commonwealth and Development Office (FCDO), the Ghana Somubi Dwumadie programme, also known as the Ghana Participation Programme, hereafter referred to as ‘the programme’:

[*S*]ought to contribute to the overall LNOB impact goal, namely that all people with disabilities, including mental health conditions are engaged, empowered, and able to enjoy improved wellbeing, and social and economic rights. (Ghana Somubi Dwumadie 2024)

The programme was led by Options Consultancy alongside partners and stakeholders from January 2020 to September 2024 and aimed to improve the lives of people with disabilities as well as those with mental health conditions. It focussed on four key areas: (1) strengthening policies and systems; (2) scaling up quality and accessible mental health services; (3) reducing stigma and discrimination; and (4) generating evidence to inform policy and practice (Ghana Somubi Dwumadie 2024; Zuurmond et al. 2025).

What made this programme unique was that it focussed on providing technical assistance to the Government of Ghana in mental health and disability and also applied user-led approaches. It also delivered quality, integrated, community-based and recovery-oriented services, empowered people with disabilities to reduce stigma and discrimination and produced evidence through research. To date, there are no research publications on the process evaluation of the strategies and methods employed to implement this complex programme. In evaluation terms, a complex programme refers to an intervention that involves multiple components – such as diverse stakeholders, cross-sectoral strategies and different implementation strategies – which collectively influence outcomes (Crane et al. 2019). The only process evaluation conducted was by the programme’s consortium partner on district mental health care plans, one of the strategies the programme used (Ae-Ngibise et al. 2024).

Taken together, these gaps underscore the need for a systematic examination of the programme’s implementation processes and stakeholder acceptability. This study presents a process evaluation of the programme, focusing on the implementation strategies guided by its Theory of Change (ToC). Specifically, we examine the feasibility and acceptability of these strategies based on the perspectives of programme partners, grantees, civil society organisations (CSOs) and government stakeholders.

## Research methods and design

### Study design

#### Theory of change

Theory of Change is an approach that describes the hypothetical steps that need to be taken to achieve planned outcomes (Breuer et al. 2016). It illustrates the relationship between the short-, intermediate- and long-term results in a causal sequence (De Silva et al. 2014). These outcomes, especially long-term outcomes or impact, may not occur until after the programme has ended, making attribution difficult (Blamey & Mackenzie 2007).

The activities or interventions required to achieve the outcomes are developed based on an existing evidence base or the knowledge of the implementers (Breuer et al. 2016). The contextual assumptions underpinning the ToC are outlined as part of the ToC process (Breuer et al. 2016) as this helps to understand what may be driving or hindering reaching the outcomes.

#### Process evaluation

The study used a process evaluation design to evaluate the programme, using the framework of the ToC. Process evaluation methods provide insight on the implementation and delivery of programmes (Skivington et al. 2021). They allow researchers and policymakers to gain a more comprehensive understanding of how these interventions work in practice. By examining the details of how programmes are put into action, we can better identify the specific mechanisms that contribute to their perceived effects (Moore et al. 2015).

#### Theory of change development

An initial ToC was informed by the funder’s call for proposals and by the experiences and priorities of programme partners. In particular, the intermediate-term, long-term and overall outcomes were informed by the funder’s wider LNOB ToC, and these did not change throughout the life of the programme.

At the end of the first 6 months of the programme (July 2020), a review of the inception period led to a number of changes to the ToC design. In particular, technical assistance to civil society was added as a key input, which the programme team now felt was needed to achieve the overall goal. The strategies being used for the ToC were further explained and expanded on, with the addition of inclusive approaches and a consideration of gender, to complement the existing user-led approaches. The inception period review relied on a range of scoping studies and desk research, supplemented by key informant interviews. The ToC, especially the assumptions, were stress tested and revised by the programme in September 2020 (Options.co.uk 2020).

Mid-way through the programme, a fuller and more detailed review of the ToC was undertaken between November 2021 and March 2022 through an iterative consultative process between programme partners. The review indicated that some elaboration and small adjustments were useful to the programme’s ToC visual and narrative. In particular, the ToC review also considered whether any adaptive actions should be taken by the programme.

The updated ToC was thereafter used as: a framework and reference document for seeing how change happens and the rationale for programme strategies and approaches; to support Monitoring, Evaluation and Learning (MEL) work by referencing where the programme logframe indicators are already measuring change across the ToC pathways; to map partners’ individual evaluation plans for assessing the programme’s results in preparation for the end of term evaluation and for wider communications on programme rationale and underpinning theory.

### Study population and sampling strategy

In-depth interviews were conducted with consortium partners, grantees, CSOs and government agencies who played a crucial role in the implementation of Ghana Somubi Dwumadie. The authors used purposive sampling to recruit study participants.

Sampling started with a mapping which allocated stakeholders into the following categories: programme partners, government stakeholders in disability, government stakeholders in mental health, CSOs receiving technical assistance and programme grant recipients. Potential interviewees were then sampled from the categories as follows:

Programme partners: There were five original programme partners who all delivered different elements of the programme, led by Options Consultancy Services Ltd., who had overall oversight. The other partners were Tropical Health, King’s College London, Sightsavers and BasicNeeds-Ghana. Each partner was written to and asked to nominate participants for interviews, and between one and two people joined each interview.

Grantees: The programme issued 21 grants across 19 organisations across three grant rounds. Because of the limited interaction with the wider programme, potential interviewees were selected based on whether they had more than one interaction with the programme such as:

- Also being a member of the Mental Health Alliance (supported by the programme).
- Having a multi-year grant.
- Being a member of the Programme Advisory Group.

Three grantees met two or more of these criteria and were invited to nominate participants for interviews, from the following organisations: Songtaba, Ghana National Association of Deaf (GNAD) and Voice Ghana (now Africa Disability Institute). In addition, a small grantee in a region without other programme work, and from the final grants round, was selected: Duapa Mothers Union. This was to secure the perspective of a smaller grantee.

Civil society organisations who received technical assistance under the programme were Ghana Federation of Disabilities (GFD) and Mental Health Society of Ghana (MEHSOG). Both were invited to interview; however, it was not possible to arrange an interview with MEHSOG because of their competing priorities.

Government stakeholders in disability were identified for interview because of having an agreed technical assistance plan in place with them. This included the Ministry of Gender, Children and Social Protection (MoGCSP) and the National Council for Persons with Disability (NCPD).

Government stakeholders in mental health were identified initially because of having a technical assistance plan, that is, with the Mental Health Authority (MHA) current and retired point persons. However, as we had also worked closely with Ghana Health Service (GHS) Mental Health Unit, they were also identified for interview to ensure a good balance between the disability and mental health sides of the programme.

Before each interview, Lyla Adwan-Kamara (L.A.-K.) made contact with participants and shared the information sheet. Dates and times were then arranged with individual stakeholders. All study participants provided voluntary informed consent to participate. In total, 32 interviews were conducted with 14 stakeholder organisations (see Table 1).

**TABLE 1:** An outline of participating stakeholder organisations (*N* = 14) and interviewees (*N* = 32).

Stakeholder organisations	*n*
**Programme partners**	
Options	3
Sightsavers	2
BasicNeeds-Ghana	2
King’s College London	2
Tropical Health	2
**Grantees**	
Duapa	6
Songtaba	2
Voice Ghana	1
Ghana National Association of Deaf	1
**Civil society organisation**	
Ghana Federation of Disabilities	1
**Government stakeholders in disability**
National Council for Persons with Disability	1
Ministry of Gender, Children and Social Protection	2
**Government stakeholders in mental health**	
Ghana Health Service	1
Mental Health Authority	6

### Data collection

Two main methods were used to collect the data, namely in-depth interviews and a document review of the programme. Documents included narrative reports from consortium partners, milestone reports and monitoring, evaluation and learning reports. L.A.-K. and L.S. conducted all the interviews in English. Interviews were conducted either in person or virtually, depending on the interviewee preference and location. The research team designed the interview guide based on the research questions and informed by the programme’s ToC, exploring perceptions of the effects of key inputs and the main strategies. The interviews then explored the validity of the key assumptions underpinning the ToC and finally explored the extent to which the interviewees felt the intermediate outcomes had been achieved. Interviews were recorded and lasted between 45 min and 1 h and 15 min.

### Data analysis

Audio files from the in-depth interviews were retrieved from the recorder, categorised into separate folders representing each stakeholder and securely stored on SharePoint with restricted access. All the interviews were transcribed by a transcription application. To protect participants’ anonymity, unique identifiers were assigned to the final transcripts for all stakeholders (Sakyi et al. 2024). Thematic analysis was then conducted using both inductive and deductive approaches (Azungah 2018) to understand stakeholders’ perspectives on acceptability and perceived effects of key strategies and inputs, challenges as well as key achievements of Ghana Somubi Dwumadie. Themes and sub-themes were developed using a two-staged approach. The deductive approach established initial categories based on the research questions outlined in the semi-structured interview guide (Sakyi et al. 2024). Subsequently, an inductive approach was employed to identify and incorporate any additional themes that emerged from the interview transcripts, ensuring a comprehensive analysis that captured all relevant insights. Analysis was done by L.S. and L.A.-K. using using NVivo (a qualitative data analysis software).

### Reflexivity statement

As researchers involved in this qualitative research, we recognise the importance of acknowledging our positionality and its potential influence on the research process. Our diverse backgrounds in public health, disability advocacy and mental health services informed our approach to exploring the acceptability and perceived impact of the programme. While this diversity enriched our perspectives, it also necessitated ongoing reflexivity to ensure that our interpretations were not unduly influenced by our professional or personal experiences. We took conscious steps to mitigate biases, such as engaging in peer debriefing and triangulating findings across multiple data sources to preserve the authenticity of participants’ voices. This reflexive process strengthened our commitment to presenting a balanced and credible account of the programme’s impact.

We also acknowledge the potential power dynamics at play in our interactions with study participants. We approached the data collection process with cultural sensitivity, ensuring participants felt respected and valued for their insights. Through open-ended interviews and participatory methods, we aimed to foster an environment where they could freely express their views on the programme’s acceptability and impact. This reflective stance guided our analysis, enabling us to remain empathetic while critically examining the findings to draw meaningful conclusions.

### Ethical considerations

Ethical approval to conduct the study was obtained from the Kwame Nkrumah University of Science and Technology (KNUST) Humanities and Social Sciences Research Ethics Committee on 07 September 2023 (Ref: HuSSREC/AP/159/VOL. 2).

## Results

### Implementation of programme’s theory of change

The programme sought to:

[*C*]ontribute to two long-term expected outcomes: (1) Quality disability inclusive, mental health treatment and rehabilitation services are sustained; (2) People with disabilities, including mental health conditions, are empowered and their rights promoted, protected, respected and realised. (Ghana Somubi Dwumadie 2024; Options.co.uk 2020)

**FIGURE 1:**
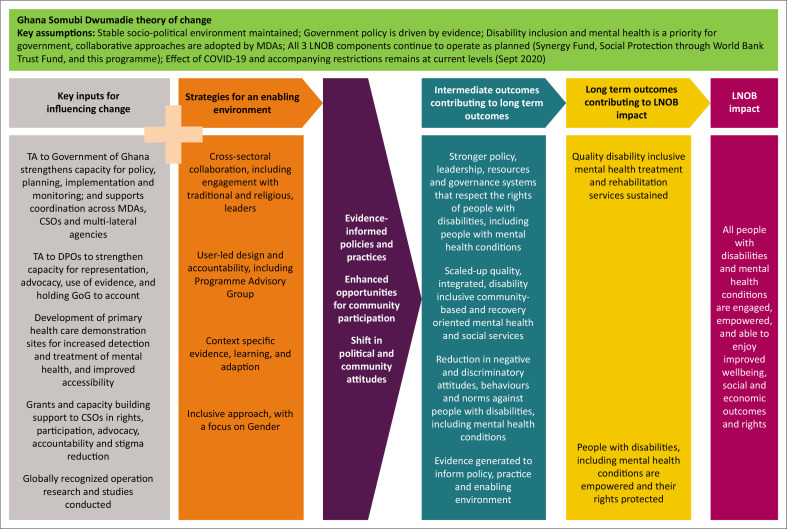
Ghana Somubi Dwumadie theory of change.

Four intermediate outcomes were outlined in the ToC (Figure 1) and sought to achieve:

- ‘Stronger policy, leadership, resources and governance systems that respect the rights of people with disabilities, including people with mental health disabilities.
- Scaled-up quality, integrated, disability inclusive community-based and recovery oriented mental health and social services.
- Reduction in negative and discriminatory attitudes, behaviours and norms against people with disabilities, including mental health disabilities.
- Evidence generated to inform policy, practice and enabling environments’.

The programme sought to achieve this by providing targeted technical support to the Government of Ghana – working through key ministries, departments and agencies – as well as offering direct assistance to organisations of persons with disabilities (OPDs); the development of primary healthcare demonstration sites for increased detection and treatment of mental health and improved accessibility of health facilities; a grant scheme to assist CSOs in addressing stigma and discrimination, while also empowering persons with disabilities, including those with mental health conditions, to advocate for their rights. Additionally, operational research was carried out to guide policy development and practical interventions in mental health and disability (Ghana Somubi Dwumadie 2024; Options.co.uk 2020).

Four key strategies were used by the programme that fed into the overall implementation: (1) ‘Cross-sectoral collaboration between different government ministries and departments, CSOs, traditional and religious leaders and the private sector working in the mental health and disability space’; (2) ‘User-led design and accountability, which sought to increase participation of people with disabilities, including mental health conditions, to ensure that services are designed and implemented in a relevant and appropriate manner, meeting the needs of service users and challenging negative attitudes’; (3) ‘Generation of context-specific evidence, learning and adaption, to support advocacy and effective service design and delivery was another strategy the programme used’; and (4) ‘Using an inclusive approach with a focus on gender, exploring a range of opportunities to elevate and enable the agency and voice of women with disabilities’ (Options.co.uk 2020).

### Acceptability of programme’s key inputs

#### Technical assistance to Government of Ghana

The programme’s ToC hypothesised that strengthening leadership and governance systems would result in more informed resource allocation, increased inclusivity in processes and mechanisms and, ultimately, the delivery of higher-quality services that cater to a broader range of needs. One of the workstreams was the assistance provided by the programme to the MHA to the establishment and operationalisation of a Mental Health Review Tribunal and Mental Health Regional Visiting Committees. Assistance provided by the programme facilitated a review of the legal framework for establishing both bodies and the agreement of the process for the nomination, vetting and appointment, inauguration, induction, orientation and training of their members.

Stakeholders indicated that the provision of technical assistance to the government by the programme was successful. One government stakeholder appreciated the added efficiency, the ability to manage time and resources better and the support in producing a significant volume of quality outputs. Stakeholders during interviews mentioned various forms of assistance, such as providing support for the implementation of the Global Disability Summit commitments. They further added that the programme has become a ‘household name’, making it easier to discuss and implement it at the community and district levels. The positive acknowledgement from various ministries and the communities’ familiarity with the programme show its broad acceptance and integration:

‘In terms of the specific support, yes, the project was able to bring on board something like a drafting of policy briefs and that was great. Not that [our institution] did not have the skill, but at least putting all together the ambience, the time, everything together to be able to bring out the briefs in the time that these numerous briefs were brought. I think it was something that we would consider a technical support. We would consider that probably would have taken much longer to have been able to do that on our own. But the totality of bringing the policy briefs, the numbers that we had, for instance, briefs on traditional and faith-based healers, briefs on psychotropic medications, various briefs, I’m not sure we’d have been able to bring them all within that space of time. So, yes, there was support along that line.’ (Government of Ghana stakeholder 3)

‘I am aware of some of these technical assistance or support to government, those that I mentioned about putting the Global Disability Summit commitment into perspective, and then informing government as to what to do, the 3% common fund and many other things that at that level, the engagement that was made at that level. And it’s also, and then quite a lot with the Mental Health Authority and all that, the tribunal that was set up. These are all the technical assistance to government, which works so well and complements the efforts of grantees at the grassroots. And it got to a point, you can see that Ghana Somubi becomes a household name.’ (Grantee 1)

#### Technical assistance to civil society organisations

The programme provided technical assistance to GFD and MEHSOG. The technical assistance included leadership training for GFD regional leaders and training of trainers on rights-based advocacy for MEHSOG. The technical assistance work with GFD led to increased ownership of advocacy and stronger leadership skills in policy-level engagements, media engagements and self-advocacy for quality services by people with disabilities. The capacity needs assessment played a key role in enhancing GFD’s organisational abilities, helping them understand their strengths and areas for improvement:

‘[*T*]here was also the technical assistance to civil society organisations, that was really helpful…considering the number of CSOs and national level NGOs that were supported even to understand basic financial policies and guidelines, issues of reporting and developing funding proposals were of help to them. There has been a huge amount of strengthening of civil society sector in Ghana for advocacy, especially within the disability and mental health sectors.’ (Consortium partner 2)

‘The capacity needs assessment was very useful because it gave us a better picture of our capacities and the gaps that needs to be addressed and following the assessment we took some actions to address some of the gaps that were identified so yes we will say it was good.’ (Civil Society Organisation 1)

#### Development of primary healthcare demonstration sites

The programme’s ToC outlined that people with disabilities, including mental health conditions, should have access to health and social services. The programme therefore sought to design a range of workstreams to demonstrate how health services can be planned and implemented with better integration of mental health diagnosis and treatment and to show how health services can be more accessible for all.

A key example was the development and implementation of integrated district mental healthcare plans. To gain a deeper understanding of the primary healthcare system and opportunities for integrating mental health services, the programme supported a situation analysis of mental health services in five demonstration districts (Weobong et al. 2023). Ghana Somubi Dwumadie subsequently worked in partnership with the GHS and the MHA to develop and implement the district mental healthcare plans in three pilot districts: Anloga in the Volta Region, Asunafo North in the Ahafo Region and Bongo in the Upper East Region (Weobong et al. 2023). As part of the implementation, the programme supported the capacity building of 84 primary healthcare providers from 15 health facilities in Mental Health Gap Action Programme (mhGAP) training. The programme also trained 17 healthcare workers from the three districts to equip them with the skills needed to train others, and they in turn trained 87 community health volunteers under the supervision of programme staff. The training equipped community health volunteers to be able to identify and refer people with mental health conditions to health centres, and general health workers were equipped to provide mental health services appropriately, including identification and referral of cases, using the WHO Mental Health Gap Action Programme (mhGAP) Intervention Guide (Keynejad, Spagnolo & Thornicroft 2021). With the support of GHS and MHA, the programme conducted supervision of the implementation of the district mental healthcare plans using the standard-operating procedures designed by the programme. A mixed-methods process evaluation on the development and implementation of district mental healthcare plans has been undertaken and reported in another study (Ae-Ngibise et al. 2024).

### Effective participatory approach

The programme’s strategy of involving multiple stakeholders was well received and viewed as effective according to interviews conducted with partners. Local stakeholders were actively engaged in crucial processes such as district selection, ToC development and the design of mental health care plans. The participatory nature contributed to a sense of ownership and buy-in from local actors and ensured that the resulting plans were well adapted to the local context, both of which were perceived as crucial for the success of the programme:

‘And I think what worked well from my perspective is having a very participatory approach from the outset, you know, involving all the stakeholders and first of all selecting the districts. And then once the districts were selected, getting the local stakeholders involved in the ToC process and designing the mental health care plans. I think that worked quite well.’ (Consortium partner 4)

### Grants and capacity building to civil society organisations

The programme adopted collaborative, co-creation and user-led approaches to designing and deploying a targeted and culturally sensitive social and behaviour change (SBC) strategy, which aimed to reduce stigma and discrimination. Since 2020, the programme has carried out three thematic grant rounds. In 2020, it introduced the COVID-19 Psychosocial Resilience Support grants. The following year, the 2021 Evidence and Effectiveness grants round supported a variety of advocacy and SBC initiatives, funding nine advocacy and SBC projects as both small and large grants, which ended in 2022 and 2023, respectively. Finally, in 2022, the programme launched the Legacy and Sustainability grants round.

The Evidence and Effectiveness grants aimed to empower people with disabilities through evidence-based approaches, training disability inclusion champions and improving support from duty bearers. Grantees employed various advocacy strategies, including interface meetings between disability groups and duty bearers, capacity building for effective advocacy and participatory user-led activities (Zuurmond et al. 2025). They also conducted research studies, disseminated findings and engaged stakeholders. Self-help groups were mobilised into advocacy networks. Grantees also trained health personnel and organised policy dialogues and awareness campaigns on mental health, emphasising engagement with government, traditional and religious institutions.

The Legacy and Sustainability grants by Ghana Somubi Dwumadie focused on strengthening small grassroots organisations to sustainably improve lives of people with disabilities, especially women. Building on previous grant experiences, four local disability and women’s rights organisations received grants from November 2022 to October 2023. Grantees were supported in developing frameworks, work plans and reports to guide and document their progress and achievements.

Interviews with stakeholders about the perceived effects of the programme’s key inputs generated themes such as capacity building and co-creation process, engagement and technical support, as well as empowerment and skill building.

### Capacity building and co-creation process

This was a two-way learning process that improved the capabilities of both the grantees and the programme implementers. They emphasised the co-creation process, as well as the collaborative and participatory nature of this approach. The local partners’ contributions to the design of the grants, materials and implementation of the work showed their integral role in the process. Stakeholders valued the input and expertise of local partners. The involvement of local partners in designing and delivering the grants demonstrated their active engagement and ownership, further supporting the acceptability of this approach:

‘I think delivering through local partners should be how we operate, though. I think that in itself as a mechanism was the right way to go about it because I think that built capacity as well as we learned from them. It was kind of that co-creation process was really, really positive and they contributed a huge amount to the design of the grants themselves, the materials, the work we delivered. So I think that was really positive. And they know their communities, so they know what can work and what can’t work. So I think that was the right mechanism.’ (Consortium Partner 1)

### Engagement and technical support

Stakeholders highlighted how the programme engaged with them as grantees and provided technical support. The programme provided support in identifying and addressing grant design areas that needed work and stakeholders valued this hands-on method, which contributed to more successful outcomes. The stakeholders appreciated that the programme did not abandon grantees after providing the grants. Instead, it offered ongoing support, including periodic trainings and technical assistance. This approach contrasts with other grant experiences where support was limited or non-existent after the grant was awarded:

‘And after we got the grants too, they didn’t just leave us like that for us to implement, but we were also given periodic trainings. They were very engaging with us, providing us with support, technical support. As to where we needed to work to make sure that. So I think the whole process and the way, the method that was used as grants, I think, is the best. And also, I think we got one grant from another country and we never knew them. We never met them until everything was done.’ (Grantee 4)

### Empowerment and skill building

The stakeholders believed that the strategy of working with local organisations on the ground was the most appropriate approach. By outsourcing implementation to local organisations, the programme facilitated skill building and empowerment among regional leaders and associations. The stakeholders noted that if Ghana Somubi Dwumadie had directly implemented the projects, there would have been no skill development or empowerment, and they would have remained mere observers. This highlights the perceived effects of the strategy in fostering local capacity and leadership. The strategy not only empowered individuals but also strengthened the organisational presence and influence of regional disability associations:

‘I think that is the most appropriate way we should have done it. Because in the disability space, the skill to do some of this work does not lie in one person. So it’s very good that you work with the people who are actually on the ground … You can’t implement something for a person with disability without involving them. Because they have been there for a very long time. That is their space. They know how to work. But because you outsource it to some of these organisations themselves, for them to implement it themselves, that was the best thing.’ (Civil society organisations stakeholder 2)

### Operations research and studies conducted

Ghana Somubi Dwumadie’s ToC hypothesised that generating and disseminating relevant evidence leads to increasing evidence-based policy and practice. The programme published 11 studies in peer-reviewed journals to build an essential evidence base for addressing healthcare disparities and influencing behavioural change. Importantly, it prioritised involving people with disabilities and mental health conditions in evidence generation, aiming to empower these groups and make the evidence more relevant and urgent.

The programme implemented a focused approach to research and studies, concentrating on five key themes: mental health and disability research agenda, grantee-led research, district mental healthcare plans, social behaviour change and self-help groups. These areas guided the programme’s comprehensive efforts in addressing mental health and disability issues through targeted research initiatives.

### Perceived effects of programme’s key strategies

The study sought to assess the perceived effects of the key strategies the programme employed to effect change. This section explores the ways in which the programme implemented the various outcomes outlined in the ToC.

#### Cross-sectoral collaboration

The programme adopted a collaborative approach to working with institutions and stakeholders who have responsibilities for upholding the rights of people with disabilities. The programme collaborated with MoGCSP, NCPD and the disability unit of the SDG secretariat under the Office of the President to bring together key stakeholders, including people with disabilities and mental health conditions, to develop a roadmap for the implementation of the 2022 Global Disability Summit disability commitments made by Ghana.

The programme made deliberate efforts to engage with traditional leaders and faith-based healers, to develop a culturally appropriate approach to engagement and influencing attitudes and practices. The programme offered various mechanisms by which grantees could engage with traditional and religious leaders, including through frequent direct consultations and interface meetings, and by involving them in the design and delivery of stigma-reduction activities, such as selecting inclusion ambassadors (Zuurmond et al. 2025).

#### Perceived effects of engaging traditional leaders

According to interviewed stakeholders, Ghana Somubi Dwumadie’s strategy of engaging traditional leaders was highly effective. One of the programme’s objectives was to reduce stigma within communities, and this was reported to be effectively communicated through the traditional leaders. The programme’s efforts resulted in concrete, measurable changes at the community level, demonstrating effective policy influence:

‘And reading just, you know, I wouldn’t say I have some knowledge across the board of the grants, but little bits and pieces that I’ve read have certainly shown that when that approach has been taken, that’s been very effective in getting those traditional leaders on board with messages around reducing stigma, with various, I don’t know, bylaws and so on. About language used and so on, all that work. I think it’s been effective and it’s, it seemed like extremely good strategy for going right to the key influences within community.’ (Government of Ghana stakeholder 2)

### User-led approaches

Ensuring that people with disabilities and their organisations were not just beneficiaries but active implementers and leaders was a key programme strategy. The user-led approach empowered and engaged people with disabilities and their representative organisations to participate in the design and implementation of key programme activities and decision-making processes that affected them. The programme instituted a Programme Advisory Group consisting predominantly of people with disabilities, including mental health conditions, to help guide programme activities and ensure that the voices of people with disabilities were heard and considered in programme design and implementation. This study sought to assess the perception of stakeholders on the perceived effects of the user-led strategy in the implementation of the programme. The following themes emerged from the interviews:

#### Effective disability-led implementation

Stakeholders contrasted Ghana Somubi Dwumadie with other projects that mention disability but fail to meaningfully involve disabled people’s organisations (DPOs). This comparison underscores the programme’s perceived effects in genuine inclusion. Some stakeholders also cited that a high percentage of advisory group members being people with disabilities demonstrated the programme’s perceived effects in ensuring representation:

‘As I explained earlier on, nothing about us without us. That’s very important. And persons with disability are the best people to lead issues about themselves. Because they live their disability, and they know … They have the experience. They know how to work around it. So, with a user-led approach … … where we have persons who are persons with disabilities themselves leading the project themselves…’ (CSO stakeholder 3)

#### Empowerment of user groups

Stakeholders underscored the perceived effects of the programme’s strategy of working with user groups and involving community members and traditional leaders from the outset. Empowering user groups to advocate for quality mental health services and including influential community members in the design and implementation phases lead to greater ownership, easier implementation and sustainability of the project. The stakeholders highlighted these practices as important and advocated for their continuation in future initiatives. This approach not only ensured the project’s relevance and acceptance but also built local capacity and leadership:

‘Where they work with user groups and try to empower them to advocate more for quality mental health services. And especially for our respective association sites to where we now design, where we deliberately included people from the communities, traditional community leaders, where we try to get them onto the operations team. So I think these were all things that we can advocate to be taken forward. When you involve the people at the beginning and make sure that they own it, then it’s easier to implement it than if you had designed it without them.’ (Consortium partner 1)

‘Well in terms of the user-led approach we saw that working very well so through our project we designed we had what we call the self-help groups who were the users themselves, the service users were strengthened into groups so that in terms of sustainability after the project end those associations will still be there. It forms some kind of social support for each other and that also worked well; It has been very effective the first one also because it was not as frequent as the rest of the three you see that the user led were involved throughout the project from the design throughout to the project.’ (Grantee 3)

### Limitations and barriers to programme implementation

#### Funding instability and unpredictability

One of the dominant themes regarding limitations of the programme was funding instability from the donor. This was a major challenge that affected the programme’s ability to plan and execute activities consistently. Interviews with stakeholders showed that the unpredictable and unstable funding environment had wide-ranging negative impacts, from issues like reduced scope and lost opportunities for dissemination to more intangible but equally important factors like eroded confidence, damaged relationships with partners and decreased team morale:

‘The negative bit is the unstable funding for the programme. When a lot of the times you are not certain whether what you are actually certain to do, you will actually do it, or not, because here and now you will hear budget cuts reduce work scope…’ (Consortium partner 2)

‘When we took off in March 2020, with all the availability of funding, there was a lot that the programme thought it could … implement in order to bring about some of these changes. But we couldn’t control the funding from the donors. They decided, year on year on, leading to the end of the programme, budget cuts here and there. Some of the activities, for instance, had to be taken off. Because of budget cuts. And there’s nothing we can do about it.’ (Consortium partner 3)

#### Gap between advocacy and implementation

One of the challenges stakeholders identified was the gap between advocacy and implementation. According to one stakeholder, there was a disconnect between the programme’s focus on advocacy and capacity building and the practical needs for service delivery on the ground. The programme not making provision for service delivery showed a significant limitation in its scope. According to stakeholders, the programme’s perceived effects were hampered by not being able to provide essential resources for mental health care:

‘The project did not make provision for service delivery. And so along the line, the challenges we came across in terms of direct service delivery that could enhance the quality of inclusive mental health care was missing because the programme could not provide those services directly in terms of medications, maybe equipment that they will need to do their work.’ (Grantee 3)

#### Lack of control over policy implementation

Another significant barrier to the programme’s perceived effects was the limited control over critical political processes. While the programme has achieved success in advocacy, the programme’s lack of direct influence over certain critical outcomes, particularly those dependent on high-level political decisions, has hindered the full realisation of the programme’s advocacy efforts:

‘Even right now, as we speak, as we have advocated and parliament has passed the law, the president is yet to sign the bill. And this is something squarely that is not under our control as a programme to be able to do, but we can find a way to push through our advocacy to get that done. But it’s something squarely that is outside our control.’ (Grantee 2)

## Discussion

The study explored the acceptability and perceived impact of the programme through a process evaluation using the programme’s ToC. The findings show that the programme’s inputs and strategies, particularly technical assistance, user-led approaches and cross-sectoral collaborations, were generally well received and contributed to disability inclusion. The programme successfully integrated mental health services into primary healthcare systems across three district demonstration sites, addressing service gaps. Furthermore, the use of grants and capacity-building initiatives empowered local civil society and disability organisations and facilitated stronger advocacy efforts. However, the programme faced challenges related to budget changes, inability to implement advocacy goals and limitations in policy implementation because of external political factors.

### Key strategies and perceived impact

The programme’s technical assistance to both government agencies and CSOs was perceived as crucial to strengthen leadership, governance and advocacy in the disability and mental health sector. Stakeholders highlighted how this support enabled the development of policy briefs, strengthened advocacy on issues like the Global Disability Summit commitments and improved organisational capacity. These findings are consistent with literature on capacity development in LMICs, which has shown that strategic technical assistance can fill institutional gaps and could accelerate systems change (Bates et al. 2014; Engelgau et al. 2018; Hansoti et al. 2019). In the disability sector, studies have highlighted the importance of building both individual and organisational capacities to ensure effective implementation of disability rights. Empowering persons with disabilities with advocacy skills, leadership training and awareness of their rights can strengthen their ability to participate meaningfully in decision-making processes and hold duty bearers accountable (Graybill et al. 2022; Kim, Hall & Jung 2021; Matsana et al. 2021). At the organisational level, strengthening the technical, managerial and advocacy capabilities of DPOs and other civil society actors is critical for sustained engagement in policy dialogues, effective programme implementation and resource mobilisation (Elbers & Kamstra 2020; Grills et al. 2020).

The programme’s implementation of district mental healthcare plans and the training of primary healthcare workers were notable attempts to integrate mental health services into Ghana’s existing primary healthcare and to narrow the treatment gap. Stakeholders viewed these efforts positively, especially in terms of increasing accessibility and strengthening early detection and referral systems through the WHO’s Mental Health Gap Action Programme (mhGAP). This aligns with global calls for integrated, community-based mental health services, particularly in LMICs where mental health systems are under-resourced (Lund, Tomlinson & Patel 2016; Ward et al. 2024). Studies have shown that integrating mental health into primary care improves service utilisation, reduces stigma and promotes continuity of care (Javed et al. 2021; Sakyi et al. 2024). Ghana Somubi Dwumadie’s demonstration sites represent an effective model for other districts to replicate in Ghana.

The grants mechanism was another effective input that empowered organisations, particularly in advocacy and social behaviour change programmes. Stakeholders appreciated the co-creation process, technical support provided after the award of grants and opportunities for skill-building. The multi-round grant structure also allowed for targeted responses to emerging issues such as COVID-19-related psychosocial needs and sustainability planning. This approach has shown to be among the best practices in participatory development, where funding goes hand in hand with ongoing mentorship and collaboration (Cann et al. 2024). The uniqueness of this was that grant-funded organisations were not passive recipients but co-implementers, contributing contextual knowledge and community trust – key elements in achieving local ownership and sustainability.

The programme’s emphasis on operational research as an input was critical to generating locally relevant evidence. The peer-reviewed publications and various internal studies addressed knowledge gaps and provided a foundation for advocacy and policy development.

A unique and widely accepted aspect of the programme was its user-led approach, which actively involved people with disabilities and mental health conditions in programme design, implementation and oversight. Mechanisms such as the Programme Advisory Group and self-help user groups fostered meaningful participation and local ownership. This aligns with the disability rights principle of ‘Nothing About Us Without Us’ and reflects best practices in participatory programme design (Zuurmond et al. 2025). Research indicates that such approaches improve service acceptability, facilitate scale-up and foster trust (Turuba et al. 2024; Watkinson, Dharmayat & Mastellos 2021). When people with lived experience are meaningfully engaged in designing, implementing and evaluating programmes, interventions are more likely to align with the actual needs, preferences and cultural contexts of the target population (Ng et al. 2024). This leads to higher levels of community trust and increases the acceptability of interventions.

Another finding was the success of cross-sectoral collaboration, which includes engagement with traditional and religious leaders. This was particularly important in addressing stigma and promoting the programme’s interventions. Stakeholders attributed the involvement of traditional leaders with influencing local norms, and even contributing to the development of bylaws related to inclusive language and behaviour. This strategy aligns with literature that highlights the role of traditional leaders in effecting social norm change, especially in areas of mental health and disability where stigma is deeply entrenched (Maiden 2021; Voors 2019). The involvement of traditional and religious leaders in stigma-reduction activities represented a novel strategy, increasing the programme’s cultural relevance and local acceptance.

However, the external funding instability and lack of control over political processes constrained its full potential. The gap between advocacy and direct service delivery highlights a significant limitation, indicating that future interventions might need to balance advocacy with tangible service provision to have a more immediate impact.

### Strengths and limitations

This study included a number of strengths. Firstly, the use of a process evaluation design rooted in the ToC allowed for a detailed assessment of the programme’s implementation and delivery. This approach provided insights into how specific strategies contributed to the programme’s outcomes, giving a clearer understanding of what worked and what did not. Secondly, the combination of in-depth interviews and document reviews ensured comprehensive data collection from a diverse range of stakeholders, including government officials, CSOs and grantees. This diversity of perspectives enriched the analysis, making the findings more robust and representative of the programme’s impact. Thirdly, the use of both inductive and deductive thematic analysis helped capture a wide range of emerging themes, allowing for a more nuanced understanding of the stakeholders’ experiences. However, the study also has limitations; one key limitation is the potential for selection bias, as the participants were purposively sampled. While this purposive sampling ensures that key stakeholders are included, it might exclude other relevant voices that could have offered different perspectives on the programme’s acceptability and perceived effects.

### Implications and recommendations

By strengthening technical assistance, user-led governance and cross-sectoral collaboration, the Ghana Somubi Dwumadie programme directly contributes to key SDG. While advocacy efforts were effective, stakeholders highlighted gaps in direct service delivery. Future programmes should balance advocacy with tangible support, such as the provision of essential mental health medications and equipment, to enhance the practical impact on communities. The effectiveness of user-led strategies in promoting inclusion and empowerment suggests the need for broader adoption of these methods in similar programmes. Ensuring the active participation of people with disabilities in programme design and implementation fosters ownership and sustainability.

Additionally, the study was conducted during the programme’s implementation phase, meaning long-term impacts could not be fully assessed. Future research should assess the long-term outcomes and sustainability of the programme’s strategies and their impact on disability inclusion and mental health services.

## Conclusion

Ghana Somubi Dwumadie has made significant contributions to disability inclusion and mental health services in Ghana, particularly through its multi-faceted approach that combined technical assistance, cross-sectoral collaboration and user-led design. This process evaluation has demonstrated that key strategies – such as providing technical assistance to government and civil society, promoting participatory governance through user-led mechanisms and fostering cross-sectoral collaboration – were perceived by stakeholders as highly effective in strengthening systems, building capacity and empowering people with disabilities. The programme succeeded in building the capacity of both government and civil society, empowering stakeholders to advocate for disability rights and integrate mental health services into primary healthcare. Its strategies, such as engaging traditional and religious leaders in stigma-reduction efforts, set it apart as a pioneering initiative in the field. However, the programme also faced notable challenges, including funding instability, the gap between advocacy and service delivery and limited control over key political processes. These challenges highlight the need for future programmes to ensure sustainable and predictable funding mechanisms and a balance between advocacy and direct service provision. Despite these hurdles, the study demonstrates that the programme made substantial strides in creating an enabling environment for disability and mental health advocacy in Ghana. Future initiatives can build on the programme’s successes while addressing its limitations to ensure deeper and more sustainable impact.
